# Hemopexin in severe inflammation and infection: mouse models and human diseases

**DOI:** 10.1186/s13054-015-0885-x

**Published:** 2015-04-15

**Authors:** Tian Lin, Dayana Maita, Sujatha R Thundivalappil, Frank E Riley, Jasmin Hambsch, Linda J Van Marter, Helen A Christou, Lorenzo Berra, Shawn Fagan, David C Christiani, H Shaw Warren

**Affiliations:** Department of Pediatrics, Infectious Disease Unit, Massachusetts General Hospital, and Harvard Medical School, 149 13th Street, Charlestown, MA 02129 USA; Department of Pediatrics, Infectious Disease Unit, Massachusetts General Hospital, 149 13th Street, Charlestown, MA 02129 USA; Department of Pediatric Newborn Medicine at Brigham and Women’s Hospital, and Harvard Medical School, 75 Francis Street, Boston, MA 02115 USA; Department of Anaesthesia, Critical Care and Pain Medicine, Massachusetts General Hospital, and Harvard Medical School, 55 Fruit Street, Boston, MA 02114 USA; Department of Surgery, MGH Burn Service, Massachusetts General Hospital, and Harvard Medical School, 55 Fruit Street, Boston, MA 02114 USA; Department of Medicine, Pulmonary and Critical Care Medicine, Massachusetts General Hospital, and Harvard Medical School, 149 13th Street, Charlestown, MA 02129 USA; Department of Pediatrics and Medicine, Infectious Disease Unit, Massachusetts General Hospital, and Harvard Medical School, 149 13th Street, Charlestown, MA 02129 USA

## Abstract

**Introduction:**

Cell-free plasma hemoglobin is associated with poor outcome in patients with sepsis. Extracellular hemoglobin and secondarily released heme amplify inflammation in the presence of microbial TLR ligands and/or endogenous mediators. Hemopexin, a plasma protein that binds heme with extraordinary affinity, blocks these effects and has been proposed as a possible treatment approach to decrease inflammation in critically ill patients.

**Methods:**

We studied mouse models of endotoxemia, burn wound infections and peritonitis in order to assess if a repletion strategy for hemopexin might be reasonable. We also measured hemopexin in small numbers of three patient populations that might be logical groups for hemopexin therapy: patients with sepsis and ARDS, patients with severe burns, and premature infants.

**Results:**

Despite severe disease, mean plasma hemopexin levels were increased above baseline in each murine model. However, plasma hemopexin levels were decreased or markedly decreased in many patients in each of the three patient populations.

**Conclusions:**

Potentially different behavior of hemopexin in mice and humans may be important to consider when utilizing murine models to represent acute human inflammatory diseases in which heme plays a role. The findings raise the possibility that decreased hemopexin could result in insufficiently neutralized or cleared heme in some patients with ARDS, burns, or in premature infants who might be candidates to benefit from hemopexin administration.

**Electronic supplementary material:**

The online version of this article (doi:10.1186/s13054-015-0885-x) contains supplementary material, which is available to authorized users.

## Introduction

Red blood cells (RBCs) are disrupted in multiple clinical settings, releasing hemoglobin and its breakdown product heme into tissues or the intravascular space. Free heme is toxic and may cause oxidizing injury and minor inflammation except in unusual circumstances [1,2]. In the presence of infection, extracellular hemoglobin and heme amplify inflammation by synergizing with microbial Toll-like receptor (TLR) agonists and non-microbial endogenous ligands, such as high-mobility group box 1 protein (HMGB1), to induce high levels of proinflammatory cytokines including tumor necrosis factor (TNF) and interleukin 6 (IL-6) [3-5].

Free hemoglobin is eliminated by binding to plasma protein haptoglobin and then transported into cells of monocytic lineage via receptor CD163 [6]. Free heme binds to a 60 kD plasma glycoprotein hemopexin (Hx) that has extraordinarily high affinity (*K*_*d*_ <1 pM) for heme, and then is transported into hepatocytes and macrophages via receptor CD91 [7]. Several lines of evidence have suggested that Hx could be beneficial in settings where infection and free heme coexist. First, Hx decreases the synergistic production of TNF and IL-6 from macrophages exposed to heme or hemoglobin together with lipopolysaccharide (LPS), *Escherichia coli*, *Staphylococcus aureus* or HMGB1 [3,5,8]. Second, Hx has been protective in mouse models of sepsis [8] and sickle cell anemia [9]. Third, elevated cell-free plasma hemoglobin [10,11] or decreased plasma Hx [8] is associated with increased mortality in sepsis. These studies support the concept that free hemoglobin and heme may drive or amplify tissue inflammation that might be suppressed by Hx, either through direct sequestering of free heme, or possibly indirectly through activation of heme oxygenase-1 (HO-1), inducing the production of anti-inflammatory CO and biliverdin [12,13]. Previous reports suggest that Hx is decreased in settings of hemolysis such as thalassemia and sickle cell disease [9,14]. However, much less is known about levels of Hx in inflammation or infection.

We measured plasma Hx concentrations in three diverse populations: patients with sepsis and acute respiratory distress syndrome (ARDS), patients with burns, and premature infants. These studies support the notion that there are some clinical settings in humans in which Hx is substantially depleted, paving the way for further studies in humans to investigate the potential therapeutic use of Hx to treat heme-amplified inflammation in infectious diseases. We also studied Hx in three mouse models: endotoxemia, burn wound infection, and peritonitis with fibrin and a blood clot. We found that mean Hx levels remained above baseline in each of the mouse models, suggesting that the study of heme-related pathophysiology in mouse models of infection may not reflect the human condition.

## Methods

### Materials

LPS from *E. coli* O55:B5 was purchased from List Biologicals (Campbell, CA, USA). *E. coli* O4:K54:H5 (CP9) was a gift from Tom Russo (University of Buffalo, Buffalo, NY, USA). Mouse hemoglobin was purified as described previously [3]. Fibrin and thrombin were purchased from Sigma-Aldrich (St. Louis, MO, USA).

### Animal models

The animal studies were approved by the Massachusetts General Hospital (MGH) Institutional Animal Care and Use Committee. Six- to eight-week-old female C57BL/6 mice (Charles River Laboratories, Wilmington, MA, USA) were used in the studies.

#### Endotoxemia

Mice were injected via tail vein with 40 μg of LPS (2 mg/kg) or 4 mg of purified mouse hemoglobin or both in a volume of 200 μl, and were bled via tail vein at 24 hours before, 1 and 4 hours after the injection. Cardiac puncture was performed 24 hours after the injection.

#### Burn mouse model of gram-negative sepsis

The mouse burn wound infection model was performed as described previously [15], followed immediately by subcutaneous injection of *E. coli* O4 at log phase into the burned area as described. Blood was collected from the tail vein at different times, except the terminal bleed, which was done by cardiac puncture. Mice were given 400 μl saline intraperitoneally after tail vein bleeding. In separate experiments, we verified that baseline mouse Hx levels in blood obtained from a group of mice that underwent cardiac puncture were not statistically different than levels from a group of mice that had blood drawn from the tail vein (see Figure S1 in Additional file 1).

#### Peritonitis model of gram-negative sepsis

This model of sepsis due to peritonitis was adapted from Ahrenholz and Simmons as described previously [15,16]. Mice were implanted with a fibrin clot with or without *E. coli* O4 as desired or with 250 μl of blood in the peritoneum. All mice were administered 0.5 ml of saline intraperitoneally immediately following surgery. Some mice were given intramuscular injection of ceftriaxone (1 mg/mouse) 2 hours after the surgery. Blood was obtained by cardiac puncture 24 hours after surgery for determination of bacteremia (by culture of dilutions on Luria Broth agar plates) and for measuring Hx and cytokines in the plasma.

### Human studies

All human studies were approved by the Partners Human Research Committee covering both Brigham and Women’s Hospital (BWH) and MGH. Blood samples were collected to make plasma by using BD vacutainer blood collection tubes with sodium heparin (Becton Dickinson and Company, Franklin Lakes, NJ, USA). All blood donors or patients except infants were aged between 18 and 40 years. All adult patients had a pre-existing central venous catheter for blood collection. Exclusion criteria included inability to obtain informed consent, pregnancy, and concurrent glucocorticoid or immunosuppressive treatment.

#### Control volunteer blood donors

Nine female and ten male healthy donors were recruited through the MGH and Partners clinical studies website. Additional inclusion criteria included a normal physical examination, a normal total hemoglobin concentration (>12.5 g/dl), and normal values of blood tests. Exclusion criteria included psychiatric disturbances, systemic disease, active smoking or less than 1 year of smoking cessation, alcohol use, any use of medications during the past 7 days, antibiotic use within 48 hours, and any type of cancer.

#### Adult patients with severe sepsis and ARDS

Blood samples were collected within 48 hours of diagnosis of sepsis and ARDS in 10 patients recruited from the MGH Surgical Intensive Care Unit. Additional inclusion criteria included clinical and laboratory diagnosis of the consensus definition of severe sepsis, ventilator dependence, and the diagnosis of ARDS made by the clinical team. Seven of the ten patients had a source of sepsis in the abdomen and one of the ten had had surgery.

#### Adult patients with severe burns

Blood samples were collected from five patients with severe burns (greater than 20% total body surface area) in the Burn Unit at MGH on the day of administration (day 1) and days 3, 5 and then each week for 3 more weeks after admission to the hospital.

#### Premature infants at various gestational ages

Cord blood was collected immediately after the birth from 149 infants born at 23 to 25, 26 to 28, 29 to 31, 32 to 34, 35 to 37, and greater than 38 weeks that were enrolled in a study of cord blood vitamin D levels at BWH, Boston, MA, USA.

#### Assays for hemopexin, heme, and cytokines

Mouse and human plasma samples were saved at −80°C and assessed for Hx concentrations using enzyme-linked immunosorbent assay (ELISA) kits from GenWay Biotech Inc. (San Diego, CA, USA), heme concentrations by heme assay kits from BioVision Inc. (Milpitas, CA, USA), and TNF or IL-6 levels by ELISA kits from R&D Systems (Minneapolis, MN, USA).

### Statistics

Data were analyzed by GraphPad Prism 6 (GraphPad Software, La Jolla, CA, USA). *P* <0.05 was considered to be statistically significant. D’Agostino-Pearson omnibus normality test was performed to assess the normality of the samples. Two-tailed unpaired *t* test was performed to compare two independent groups of samples of ARDS patients. To compare more than two samples, we performed analysis of repeated measures or ordinary one-way analysis of variance (ANOVA) for Gaussian distributed samples and Kruskal-Wallis tests for non-Gaussian distributed samples, respectively. To do pairwise comparisons, Donnett’s and Tukey’s tests were used for Gaussian and Dunn’s tests were used for non-Gaussian distributed samples. Pearson correlation analysis was performed for correlation studies of bacteremia with plasma IL-6 or Hx concentrations in the mouse peritonitis model.

## Results

### Hx in mouse models

#### Endotoxemia

**Figure 1:**
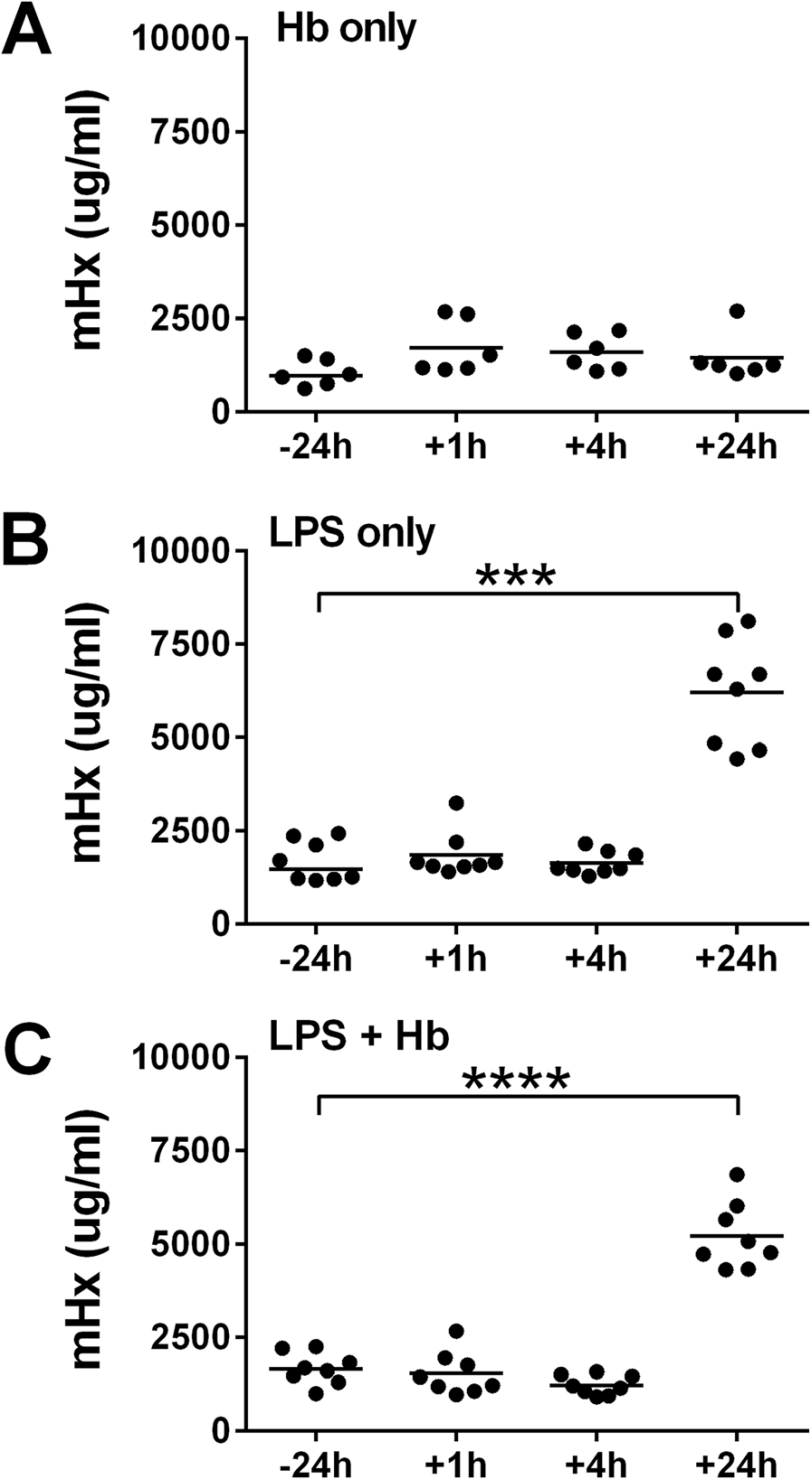
Plasma hemopexin concentrations in mouse endotoxemia model. C57BL/6 mice were injected intravenously with **(A)** hemoglobin (Hb) 4 mg/mouse (n = 6), or **(B)** lipopolysaccharide (LPS) (2 mg/kg) (n = 8) or **(C)** both (n = 8) via tail vein. Mouse hemopexin (mHx) concentrations were measured 24 h before or 1 h, 4 h, or 24 h after the intravenous injection. Data are analyzed by repeated measures one-way analysis of variance (ANOVA). Horizontal lines represent means. ^***^ *P* <0.001, ^****^ *P* <0.0001, −24 h vs. +24 h.

Mice were injected with hemoglobin, LPS, or a mixture of both. Hemoglobin alone did not induce TNF. LPS induced the production of TNF, which was significantly enhanced by injection with LPS (data not shown), as has been previously described [17]. Hemoglobin alone did not have an effect on the levels of Hx in the plasma, but LPS with or without hemoglobin significantly increased Hx concentrations (Figure 1).

#### Burn mouse model with gram-negative sepsis

**Figure 2:**
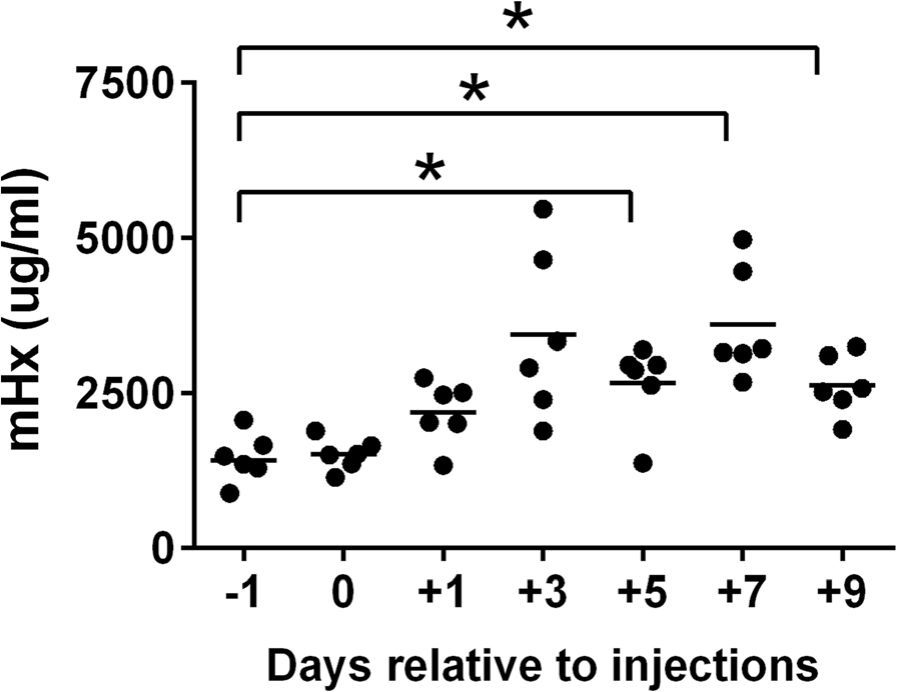
Plasma hemopexin concentrations in mouse burn model with infection. C57BL/6 mice (n = 6) were subjected to burn by boiled brass bars on the dorsal surface for 30 seconds under anesthesia, followed by a subcutaneous injection of *E. coli* O4 (10^6^ cfu/mouse) into the burned area. Mouse hemopexin (mHx) concentrations were measured 1 day before or 1, 3, 5, 7 and 9 days after the burn. The time of the burn is considered as ‘0’ time. *P* <0.01 for the whole experiment analyzed by repeated measures one-way analysis of variance (ANOVA). Donnett’s test was done for comparisons to the control. Horizontal lines represent means. ^*^ *P* <0.05, − 1 day vs*.* +5, or +7, or +9 days.

We studied Hx in a mouse burn wound infection model as described previously [15,16]. Plasma Hx concentrations increased by day 1 and became significantly increased on days 5, 7 and 9 after the burn and infection with *E. coli* O4 10^6^ cfu/mouse (Figure 2). In mice with severe infections (*E. coli* O4 10^7^ cfu/mouse injected), Hx was increased by day 1 and remained above baseline despite high bacteremia and high levels of IL-6. Eighty percent of these mice died. Mice with the lowest Hx levels survived the shortest time (data not shown).

#### Peritonitis model of gram-negative sepsis

**Figure 3:**
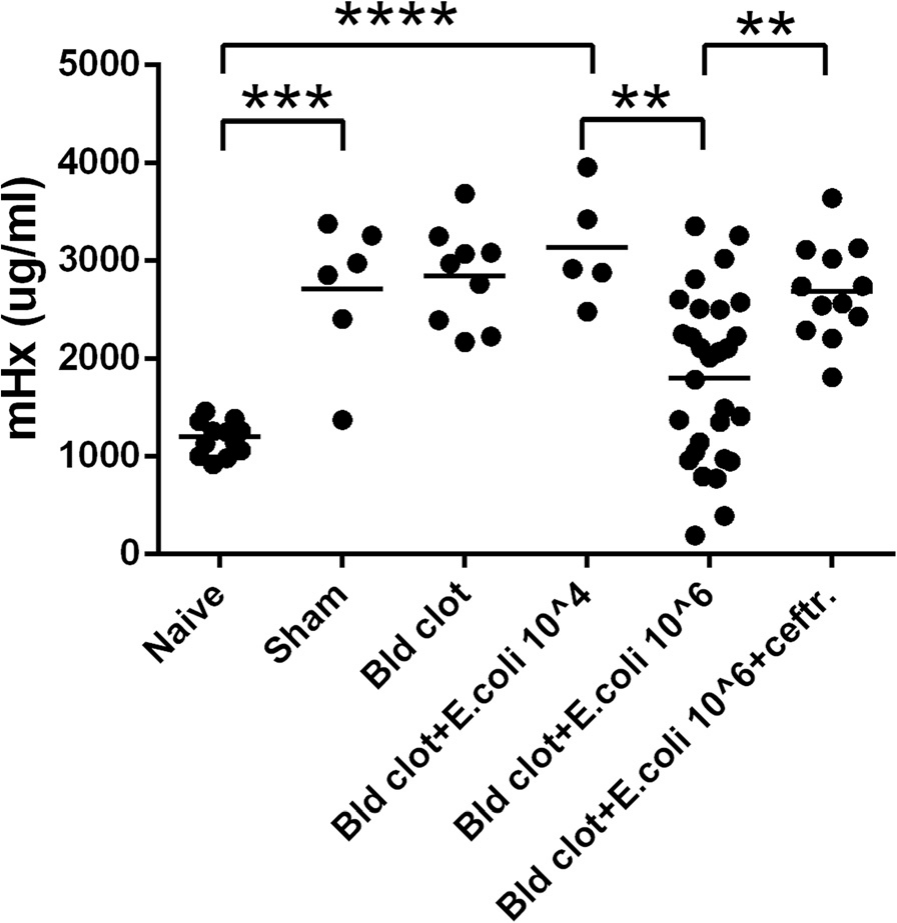
Plasma hemopexin concentrations in mouse peritonitis model with fibrin clot. C57BL/6 mice were subjected to intraperitoneal implantation of fibrin clots only (sham, n = 6) or with blood clots (Bld, n = 9) or with *E. coli* O4 10^4^ cfu/mouse (n = 5) or 10^6^ cfu/mouse (n = 37, 8 mice died) or *E. coli* O4 10^6^ cfu/mouse with ceftriaxone (ceftr.) (1 mg/mouse) (n = 12) injected intramuscularly 2 hours after the laparotomy. Mouse hemopexin (mHx) concentrations were measured in naïve mice (n = 12) or in treated mice 24 hours after the laparotomy. Data are analyzed by one-way analysis of variance (ANOVA) and Tukey’s test was done for pairwise comparisons. Horizontal lines represent means. Data were from nine independent experiments. ^**^ *P* <0.01; ^***^ *P* <0.001; ^****^ *P* <0.0001.

**Figure 4:**
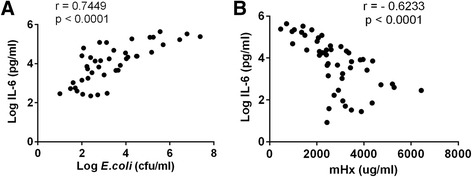
Correlation of IL-6 concentrations with bacteremia or mouse hemopexin concentrations. C57BL/6 mice (n = 48) were subjected to intraperitoneal implantation of *E. coli* O4 10^4^ to 10^7^ cfu/mouse. Cardiac puncture was performed 24 hours after the laparotomy to collect blood for interleukin 6 (IL-6) and hemopexin measurements. Blood samples were also diluted and spread on Luria Broth agar plates and the colonies were counted after overnight incubation at 37°C. Bacteremia was calculated and presented as cfu/ml. Pearson correlation analysis was performed. **(A)** Log IL-6 (pg/ml) vs. Log *E. coli* (cfu/ml) (6 out of 48 mice without bacteremia were not included); **(B)** Log IL-6 (pg/ml) vs*.* mouse hemopexin (mHx) (μg/ml). Data were from nine independent experiments.

In the peritonitis model with implantation of a peritoneal blood clot with or without *E. coli,* Hx levels increased at 24 hours by two- to threefold compared with controls after the sham surgery, and these levels were not different from those mice with surgery plus implantation of a blood clot with or without *E. coli* 10^4^ cfu/mouse (Figure 3). Increasing the bacterial inoculation in the blood clot to10^6^ cfu/mouse significantly decreased the Hx levels compared to the clot with 10^4^ cfu/mouse, although even with these high bacterial inocula the median Hx level remained above those of naïve mice. The decrease in Hx levels was not seen if the animals were administered ceftriaxone 2 hours after the surgery. We also found that bacteremia was strongly and positively correlated with IL-6 concentrations (Figure 4A). However, IL-6 levels were negatively correlated with Hx levels (Figure 4B).

### Hx in human conditions

#### Sepsis and ARDS

**Figure 5:**
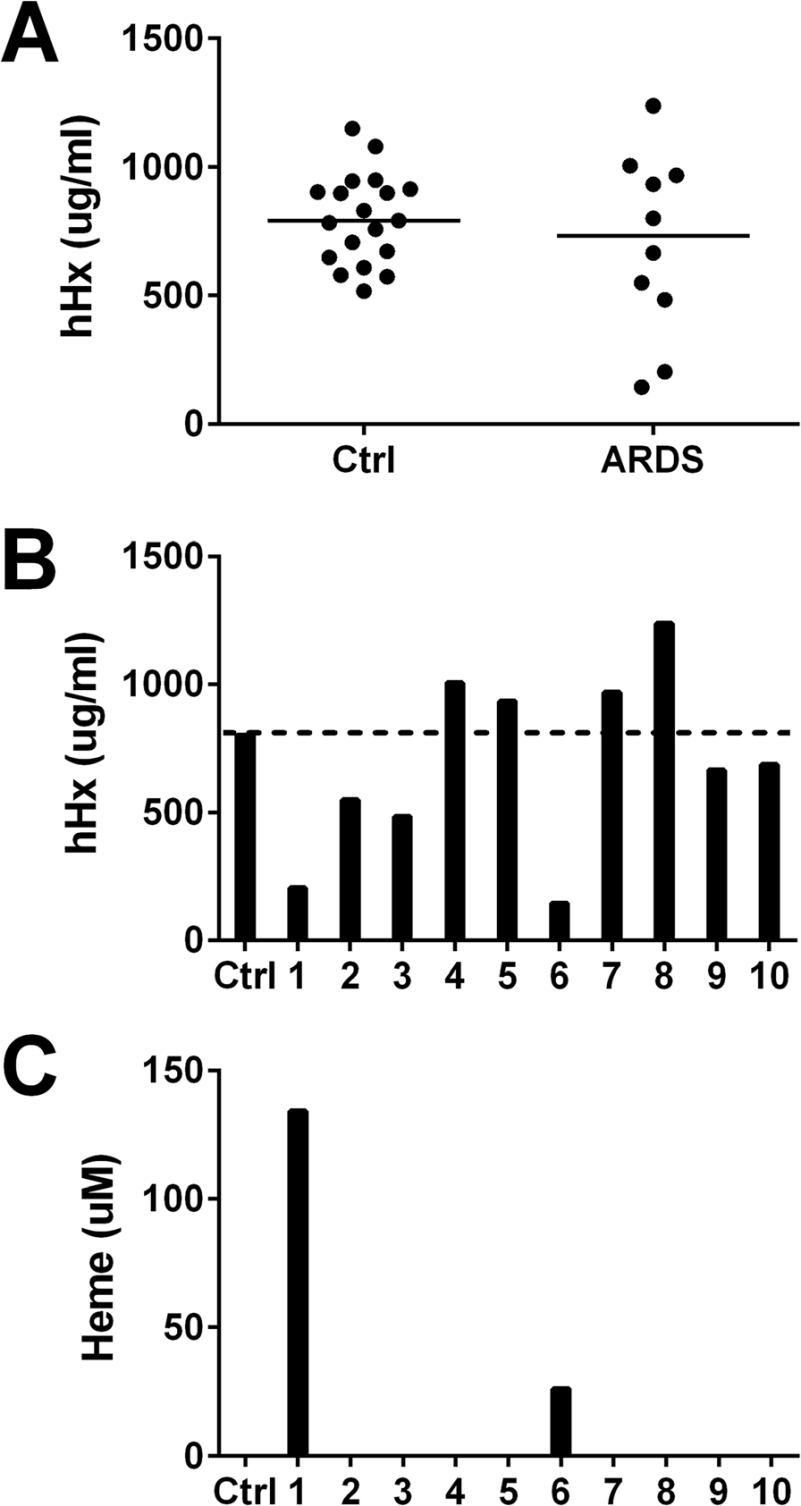
Plasma hemopexin and heme concentrations in patients with sepsis and acute respiratory distress syndrome (ARDS). Human hemopexin (hHx) concentrations were measured in 10 patients with sepsis and ARDS (1 to 10). **(A)** Horizontal lines represent medians. Data were analyzed by unpaired *t* test. There is no significant difference between the two groups. **(B)** and **(C)** Bars represent hemopexin **(B)** and heme **(C)** in the plasma from individual patients and the mean of 19 healthy donors as the control (Ctrl, dotted line).

Hx concentrations varied greatly in patients with sepsis and ARDS (Figure 5A). Two patients had very low levels of Hx compared to the control healthy donors (Figure 5B), and these two patients had elevated plasma heme concentrations (Figure 5C).

#### Burn patients

**Figure 6:**
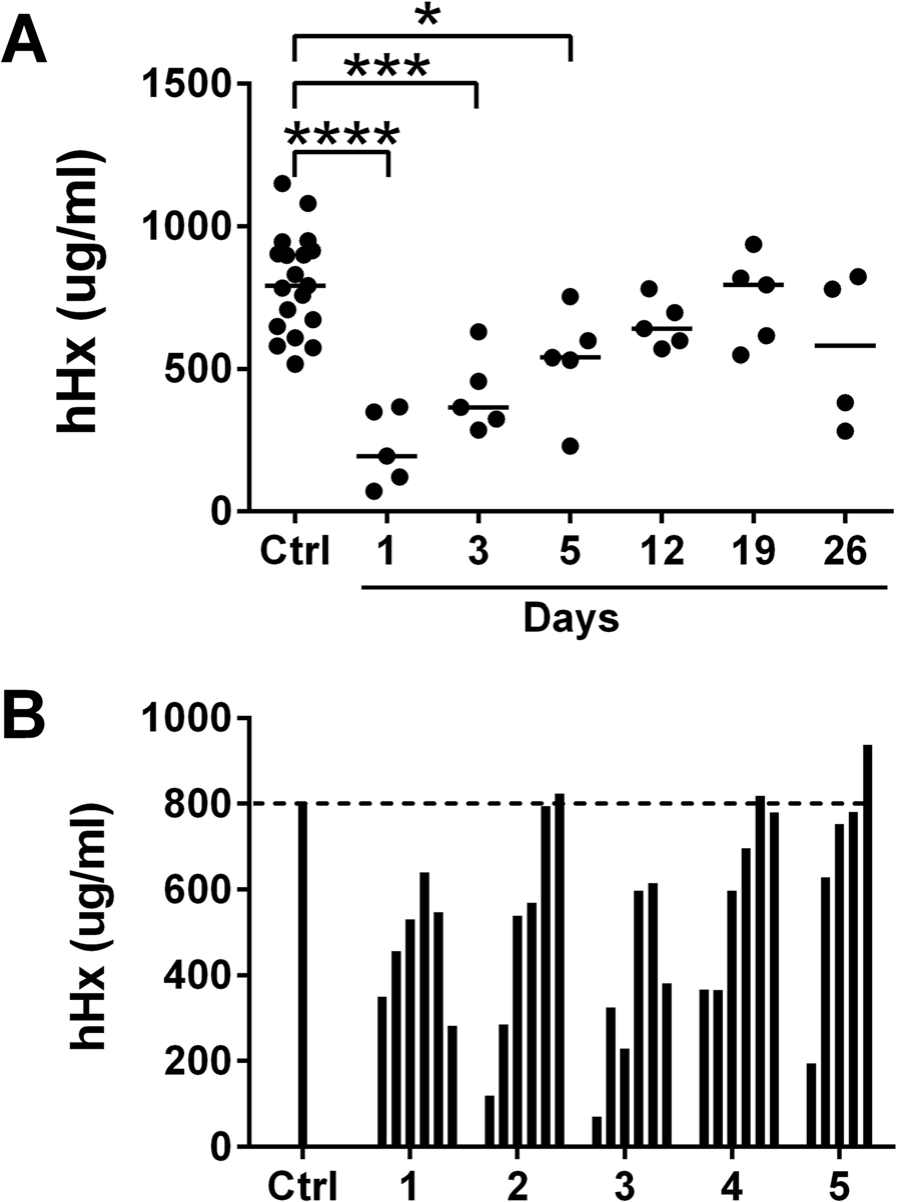
Plasma hemopexin concentrations of burn patients. Blood was drawn from 5 patients with severe burns at intervals up to 4 weeks on different days after admission to the hospital. Concentrations of human hemopexin (hHx) were measured in the plasma. Samples from 19 healthy donors were used as controls (Ctrl). **(A)** Data were analyzed by one-way analysis of variance (ANOVA) and Donnett’s test was done for comparisons to the control. Horizontal lines represent medians. ^*^ *P* <0.05; ^***^ *P* <0.001; ^****^ *P* <0.0001. **(B)** Bars represent the data from the individual patients (1 to 5) at the times in the same order shown in **A**. Dotted line represents the mean hHx concentration in the control sample (Ctrl).

During the first week after admission to the burn center, Hx concentrations in patients with severe burns were significantly lower than levels in the control healthy blood donors. Levels slowly increased over the next 3 weeks (Figure 6A). Hx levels decreased again in two patients (patient 1 and 3) during episodes of sepsis (Figure 6B).

#### Premature infants

**Figure 7:**
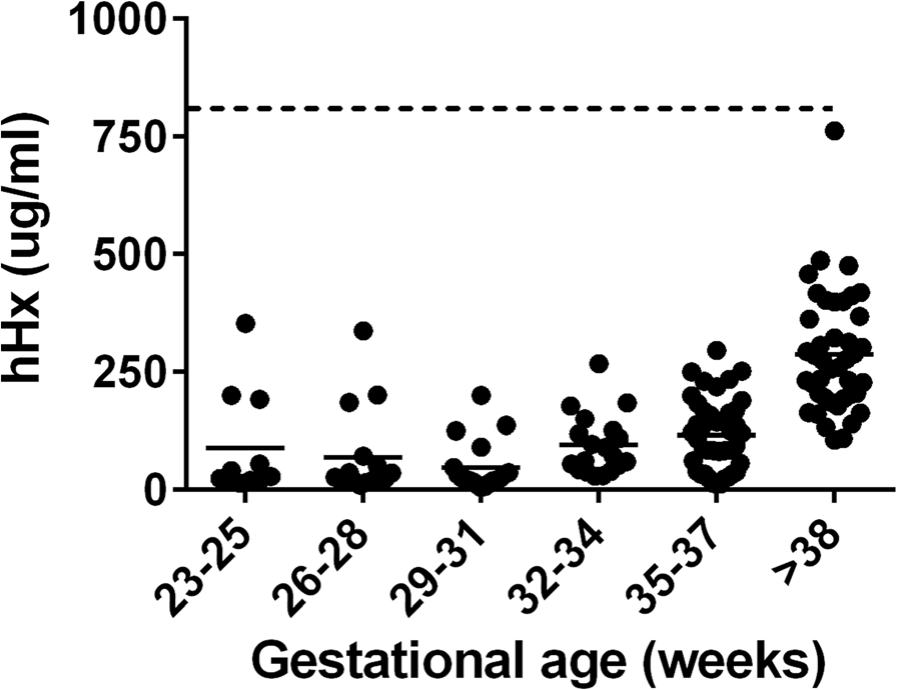
Plasma hemopexin concentrations in infants. Hemopexin concentrations were measured in the cord blood of 149 infants at different gestational ages shown (23 to 25 weeks, n = 11; 26 to 28 weeks, n = 16; 29 to 31 weeks, n = 19; 32 to 34 week, n = 19; 35 to 37 weeks, n = 42; >38 W, older than 38 weeks, n = 42). Dotted line represents the mean human hemopexin (hHx) concentration in healthy adult donors (800 μg/ml). Data were analyzed by Kruskal-Wallis test and Dunn’s test was done for paired comparisons. Horizontal lines represent means. *P* <0.0001, infants >38 W vs*.* each group of infants at different gestational ages <38 W.

Hx concentrations in the cord blood of 149 infants at different gestational ages were much lower than in the 19 normal adult blood donors studied (shown as a mean by the dotted horizontal line) (Figure 7). Premature infants at different gestational ages had significantly lower Hx levels than those in infants delivered at gestational ages older than 38 weeks (*P* <0.0001).

## Discussion

The findings that heme and hemoglobin synergize with TLR agonists, bacteria and HMGB1 to amplify inflammation and this synergy is suppressed by Hx [3,5,8] raise the possibility of using Hx as a treatment in settings where free heme may be present in plasma or tissues in the presence of infection, especially in situations in which endogenous Hx is depleted. Here we investigated Hx concentrations in three different groups of patients that might be logical early target populations for clinical trials. The goal of these studies was to evaluate the range of Hx concentrations in each population to see if replenishment might be reasonable, rather than to attempt to correlate levels with outcome, which would require much larger studies.

We first studied patients with sepsis and ARDS. There is evidence of increased RBC and hemoglobin in intra-alveolar space in these patients [18], and in mice that received intratracheal injection of LPS [19]. We found that two patients had extremely low Hx levels (<250 μg/ml) and elevated heme levels, and the patient with the highest heme level died. The other patients had varied Hx levels. There have been two prior studies of Hx in patients with sepsis of all causes. One study found that decreased Hx correlated with increased mortality [8]. A different study also found that nonsurvivors had significantly lower Hx levels than survivors [20]. These findings are consistent with two recent studies that reported that elevated plasma hemoglobin correlates with mortality in patients with sepsis [10,11]. Patients in the intensive care unit who had elevated hemoglobin but without infection did not have increased mortality [11]. We are unaware of prior studies of Hx in patients with ARDS. Although our results suggest that Hx levels in patients with severe sepsis and ARDS vary markedly, a much larger study in this population will be needed to determine if Hx is predictive of poor outcome in patients with ARDS.

We also studied levels of Hx in five patients with severe burn injury. We found that Hx was depleted in all patients on admission, and that levels in all patients rose over a period of weeks but never above normal values during the hospital course. Hx levels in two patients decreased again during episodes of sepsis. We hypothesize that heme liberated into the tissues and blood during the acute burn injury led to temporary consumption. This is the first report of Hx levels in patients with burns.

The third clinical setting that we evaluated was premature infants. Very low birth weight infants have low levels of many plasma proteins, receive frequent blood transfusions, and have greater risk of infections than pediatric patients or adults. Premature birth is associated with a high rate of secondary morbidities, including acute and chronic lung disease, necrotizing enterocolitis, retinopathy, intraventricular hemorrhage, and intracranial white matter injury, virtually all of which have been linked with inflammation. Furthermore, the evolution of chronic lung disease and retinal changes of retinopathy of prematurity (RoP) are known to be associated with oxidant injury, and the risk of RoP appears to be reduced by restricting blood transfusion [21]. These and other complications associated with hemorrhage or hemolysis are likely to be related to or worsened by heme toxicity [22,23]. We found that all premature infants had strikingly low levels of Hx that gradually rose with gestational age. However, even at gestational ages greater than 38 weeks, all infants had much lower levels than adults. These findings are consistent with, and extend, the prior report of Kanakoudi *et al.* who reported in 1995 that Hx as measured by nephelometry was low in premature infants [24].

Our main finding that Hx is decreased in many patients in each setting we studied, in some cases markedly, suggest that a subset of patients with low Hx might benefit from Hx repletion as a therapy to decrease inflammation in infection amplified by free heme and hemoglobin. Most previous drugs that have been studied in attempts to treat infection-induced inflammation by blocking a wide variety of different aspects of the immune system have the potential side effect of immunocompromise [25]. One might expect that a repletion strategy with a plasma protein such as Hx would have low toxicity, and blocking excess heme seems unlikely to cause a deficit in immune function.

A mouse model system would be helpful to study details of roles of heme and Hx in infection-induced inflammation, as well as to aid in the development of Hx preparations for human use. We investigated mouse models for endotoxemia, burn injury, and sepsis induced by peritonitis because we were unable to find murine models that convincingly mimic aspects of sepsis-induced ARDS and premature infants. Concentrations of hemopexin were maintained at or above the baseline level in most mice. Inoculation of very high numbers of bacteria resulted in decreased levels of Hx in some mice in the peritonitis model relative to those in sham mice, but mean levels remained above baseline. These findings are consistent with results obtained by Larsen and *et al*., that in a mouse sepsis model of cecal ligation and puncture (CLP), Hx concentrations were decreased relative to mice that had sham surgery, but were not much different than initial baseline levels [8]. The findings are also consistent with those reported by Spiller *et al.* that Hx is increased after mild CLP but not much different from baseline after severe CLP at later time points [26]. There is an increasing controversy as to how well mouse models of inflammation mimic human conditions, including in sepsis and burns, and a recent article proposed that it may be prudent to verify whether genes or gene products behave in a similar manner in mice and humans [27]. Some limitations in our study that could be factors in the appropriate comparison of our results in mice to humans include that we studied a single strain and gender of mice, that we studied mice of 6 to 8 weeks old compared to young adult patients, and that endotoxin challenge and peritoneal infection may not reflect sepsis causing ARDS.

Hx is an acute phase protein in several mammals, including mice, but is less of an acute phase protein in humans [28,29] so that inflammatory stimuli such as infection or tissue injury lead to only slightly elevated levels in humans. IL-6 is the main cytokine that induces the synthesis of Hx in hepatocytes during the acute phase response in mice [30]. We found that levels of IL-6 were positively correlated with the bacteremia as shown in other studies [31]. However, IL-6 was negatively correlated with Hx levels in mouse models in our studies, possibly due to hepatic dysfunction or the increased consumption of Hx by binding to increased free heme that outstripped production, or both.

## Conclusions

Recent reports that plasma cell-free hemoglobin is associated with poor outcomes in sepsis [10,11], together with data that hemoglobin and heme are synergistic with microbial ligands to cause overactive inflammation [3], support the hypothesis that hemoglobin or secondarily released heme could be causative of immunopathology, and in turn that Hx might be therapeutic [8,9]. The data reported here that some adult patients with ARDS, most patients with burns, and most premature infants, have very low levels of Hx suggest the possibility that there may be inadequate neutralization or clearance of heme in some of these patients. Our results also suggest that it is difficult to mimic the human Hx dynamics in mouse inflammatory models and therefore it may be necessary to search for a different species to model the heme axis in human infections. A different approach might be to perform association studies in each of the three populations studied here to show plausibility. Then, if low Hx is associated with increased morbidity or mortality, it might be reasonable to show that a Hx product is safe, and then proceed directly to a treatment study using patients selected for high extracellular heme or low Hx levels.

## Key messages

- Plasma hemopexin concentrations are decreased or markedly decreased in some patients with sepsis and acute respiratory distress syndrome, some patients with severe burns, and most premature infants. These results support the concept that there is a subset of patients in each population who might benefit from hemopexin repletion.
- Mean plasma hemopexin levels are increased above baseline in mouse models of endotoxemia, burn wound infection, and peritonitis-induced sepsis.
- The differing behavior of hemopexin in humans and mice in burns, and perhaps also in other inflammatory conditions, suggests that utilization of mouse models to study heme-related inflammation in humans should be done with caution.

## Additional file

Additional file 1: Figure S1. Mouse hemopexin concentrations are not significantly different between plasma prepared from cardiac puncture and from tail vein bleeding. Blood was collected by cardiac puncture (CP, n = 15) or by tail vein bleeding (n = 32) from 6- to 8-week-old naive C57/BL6 mice. Mouse hemopexin levels were measured in the plasma. Data are inclusive from three pooled experiments for CP and from three pooled experiments for tail vein bleeds and are analyzed by unpaired *t* test. Horizontal lines represent medians. There is no significant difference between the CP and tail vein groups.

## Abbreviations

ANOVA: analysis of variance
ARDS: acute respiratory distress syndrome
BWH: Brigham and Women’s Hospital
CLP: cecal ligation and puncture
ELISA: enzyme-linked immunosorbent assay
HMGB1: high-mobility group box 1 protein
HO-1: heme oxygenase-1
Hx: hemopexin
IL-6: interleukin 6
LPS: lipopolysaccharide
MGH: Massachusetts General Hospital
RBC: red blood cell
RoP: retinopathy of prematurity
TLR: Toll-like receptor
TNF: tumor necrosis factor
